# Significance of siderophore-producing cyanobacteria on enhancing iron uptake potentiality of maize plants grown under iron-deficiency

**DOI:** 10.1186/s12934-024-02618-4

**Published:** 2025-01-04

**Authors:** Mandees Bakr Brick, Mervat H. Hussein, Amr M. Mowafy, Ragaa A. Hamouda, Amr M. Ayyad, Dina A. Refaay

**Affiliations:** 1 Department of Botany, Faculty of Science, Mansoura University, Mansoura, 35516 Egypt; 2 Department of Biological Sciences, Faculty of Science, New Mansoura University, New Mansoura City, Egypt; 3 Department of Microbial Biotechnology, Genetic Engineering & Research Institute, Sadat City University, Sadat City, Egypt

**Keywords:** *Synechococcus mundulus*, Hydroxamate siderophore, NMR, CAS liquid assay, CCD, Iron-deficiency, Maize

## Abstract

**Background:**

In response to iron deficiency and other environmental stressors, cyanobacteria producing siderophores can help in ameliorating plant stress and enhancing growth physiological and biochemical processes. The objective of this work was to screen the potential of *Arthrospira platensis*, *Pseudanabaena limnetica*, *Nostoc carneum*, and *Synechococcus mundulus* for siderophore production to select the most promising isolate, then to examine the potentiality of the isolated siderophore in promoting *Zea mays* seedling growth in an iron-limited environment.

**Results:**

Data of the screening experiment illustrated that *Synechococcus mundulus* significantly recorded the maximum highest siderophore production (78 ± 2%) while the minimum production was recorded by *Nostoc carneum* (24.67 ± 0.58%). Therefore, *Synechococcus mundulus* was chosen for the beneficiary study and the intended agricultural application. Siderophore-type identification tests proved that *Synechococcus mundulus* produced hydroxamate-type. The response surface approach was successful in optimizing the conditions of siderophore production in *Synechococcus mundulus* with actual values for maximum biomass (387.11 mg L^− 1^) and siderophore production (91.84%) higher than the predicted values. The proton nuclear magnetic resonance (^1^H NMR) analysis data and the Fourier transformer-infrared spectrum analysis (FT-IR) signify the hydroxamate nature of *Synechococcus mundulus* isolated siderophore. *Zea mays* seedlings’ growth response in the hydroponic system was significantly stimulated in response to supplementation with *Synechococcus mundulus* siderophore in the absence of iron compared to plants grown without iron and the positive controls. Additionally, the contents of chlorophyll a, chlorophyll b, carotenoids, total carbohydrates, and total protein were all surpassed in siderophore-treated plants, which is expected due to the increased iron content.

**Conclusions:**

The results introduced in this study highlighted the significant potential of *Synechococcus mundulus*-derived siderophore in stimulating *Zea mays* physicochemical growth parameters and iron uptake. Findings of this study present novel visions of cyanobacteria producing siderophores as an ecofriendly alternative candidate to synthetic iron chelators and their role in plant stress management.

## Background

Iron is one of the most significant heavy metals affecting plant growth and development. Iron plays a crucial role in plant redox reactions of different physiological processes, several enzymatic activities, chlorophyll biosynthesis and as a co-factor in the synthesis of many plants’ hormones [1, 2]. Soil salinization, high pH, and unsuitable land usage can all lead to iron deficiency causing tissue necrosis, root blackening, and an overall reduction in plant growth, productivity, and nutritional quality [3–5].

Although plant roots release siderophores to regulate iron levels for metabolic processes in iron-stressed soils, this does not achieve an optimal iron level [6–8]. Therefore, cyanobacterial siderophores could be used to boost plant growth and yield, offering a sustainable agricultural option that seeks ecologically safe alternatives to chemical-based fertilizers [9, 10]. Cyanobacteria siderophores serve a variety of roles including biocontrol, medicinal application, protection against oxidative stress, and agriculture [11, 12].

Several studies revealed that soil inoculation with bacterial siderophores provides plants with iron to boost their development and health at limited iron bioavailability [13–15]. For instance, *Bacillus aryabhattai* siderophore was highly effective in rice cultivation, increasing crop production by 60% under non-saline conditions and by 43% under saline conditions [16]. Additionally, increased iron content in the tissues of *Arabidopsis thaliana* was observed following the uptake of the Fe–pyoverdine complex produced by *Pseudomonas fluorescens* C7 [17].

Cyanobacteria produce siderophores as a survival strategy in iron-limited habitats such as lakes, rivers, and oceans as iron is present in nanomolar concentrations whereas siderophores are highly effective as iron chelators [16, 18, 19].

Cyanobacteria-derived siderophores having numerous structures such as hydroxamates from *Anabaena* sp., *Agmenellum quadruplicatum*, *Anabaena catenula*, *Synechococcus* sp., *Oscillatoria tenuis* and *Phormidium autumnale* in addition to catecholates from *Anabaena cylindrica*, *Sprirulina platensis*, *Sprirulina maxima*, *Synechococcus* sp. and carboxylates, and alpha-hydroxycarboxylates from other cyanobacterial strains, all featuring two oxygen donor atoms within either linear or cyclic structures [20–22].

While iron deficit state stimulates siderophore formation by cyanobacteria, additional abiotic variables like nitrogen source, iron concentration, pH, temperature, and presence of heavy metals also influence siderophore production in cyanobacteria [23, 24]. Several investigations have demonstrated that *Anabaena cylindrica* produces high siderophores yield when exposed to high FeCl₃ (20–100 µM) [25]. However, *Phormidium* sp. produces a large amount of siderophore under iron-deficient conditions. Similarly, *Anabaena oryzae* was discovered to produce approximately 90% of its siderophores in iron-depleted environments [26]. Also, siderophore synthesis is highly sensitive to pH, which affects iron solubility and availability. According to the findings of Tailor and Joshi [27], the maximum siderophore production of *Pseudomonas fluorescens* strain was achieved at neutral pH where iron is present in insoluble form. The cyanobacterium *Anabaena oryzae* demonstrated the highest siderophore production (88.52%) at pH 7 and 54.47% at pH 8 [26]. In contrast, *Phormidium* sp. reached 70% siderophore production at pH 8 compared to 64.5% at pH 7 [28].

Furthermore, the type of nitrogen source serves a critical function in siderophore synthesis by cyanobacteria. For instance, *Anabaena oryzae* produced 95.35% siderophore production with NO₃^⁻^ as the nitrogen source and 93.34% with atmospheric N₂. However, the other nitrogen sources NH₄^⁺^ and glutamine, did not significantly stimulate siderophore production [26].

Taking the above information into consideration, the current study’s objectives are to screen the potentiality of some cyanobacterial species for siderophore production under iron-deficient conditions, optimize the various growth variables involved in the growth nutrient medium for maximum siderophore production, then investigate the potential of the isolated siderophore for enhancing maize growth attributes, chlorophylls content and biochemical responses in hydroponic-iron limited-based culture.

## Materials and methods

### Chemicals

Chrome azurol sulfonate (CAS) and hexadecyltrimethyl ammonium bromide (HDTMA) were supplied by Sigma chemical company. Other chemicals used in this study supplied by Egypt’s El Gomhouria Company for Trading Chemicals and Medical Appliances, and they were of analytical grades.

### Cyanobacterial strains

Four different cyanobacterial strains were tested for siderophore production in this study. Isolates were obtained from the Microalgal Collection (MUMC) of Algae Biotechnology and Water Quality Lab, Faculty of Science, Mansoura University, Egypt. The isolates were identified according to [29] as *Synechococcus mundulus* Skuja, *Arthrospira platensis* Gomont, *Pseudanabaena limnetica* (Lemmermann) Komárek, and *Nostoc carneum* C. Agardh ex Bornet & Flahault. Cyanobacterial cultures were maintained on BG11 media [30] at 25 ± 2 °C under illumination of 100 mol. m^-2^ s^-1^ with light: dark photoperiod of 16: 8 h.

### Growth and siderophore production under iron limitation

To recognize the siderophore-producing cyanobacteria, a siderophore-inducing BG11 medium [31] was prepared by substituting ferric ammonium citrate with FeC1_3_.6H_2_O and excluding citric acid. To assure iron-restricted cells, FeC1_3_.6H_2_O was supplied at a final concentration of 0.0051 µM, whereas the iron-sufficient control was 0.42 µM FeCl_3_. pH was adjusted at 7.4 using Tris-HC1 and deionized water was used in the preparation to ensure the absence of any residual metals. The test algae were grown in 500 mL Erlenmeyer flasks with 200 mL of BG 11 iron-limited medium inoculated with 20 mL of a 5-day-old algal culture. The cultures were incubated for 24 days at 25 °C with a constant illumination level of 100 µmol m⁻² s⁻¹. Dry biomass and siderophore production were evaluated every two days throughout the incubation. Cyanobacterial biomass was harvested by centrifugation at 2683 × *g* for 20 min. The supernatant was then utilized to identify siderophore synthesis. The pellets were dried at 65 °C to maintain a consistent weight. To remove any residues of iron, all the experiment’s glassware was soaked and prewashed with hydrochloric acid (6 M HCl).

### Quantitative estimation of siderophores

#### Chromazurol sulfonate (CAS) liquid assay

The CAS colorimetric test was utilized to confirm and estimate the siderophore production [32]. This assay depends on the competitive removal of Fe from the CAS complex by siderophore resulting in a color change from blue to orange, allowing colorimetric measurement of the blue color loss indicating the production of siderophores [33]. CAS assay solution was freshly prepared by dissolving 60.5 mg of chrome azurol sulfonate (CAS) in 50 mL de-ionized H_2_O, then combined with 10 mL Fe^**+** 3^ solution (1mM FeCl_3_.6H_2_O dissolved in 10 mM HCl). This solution was added gradually while stirring to the hexadecyltrimethylammonium bromide (HDTMA) solution (72.9 mg/40 mL de-ionized water with adjusted pH at 6.8 by tris HCl), whereas a dark blue coloration was developed. Finally, each culture supernatant (0.5 mL) was mixed with 0.5 mL CAS reagent and then incubated at room temperature for 1 h and the optical density was measured at 630 nm. The yield of siderophore was calculated as siderophore units (%) according to this equation:

$$\:\mathbf{S}\mathbf{i}\mathbf{d}\mathbf{e}\mathbf{r}\mathbf{o}\mathbf{p}\mathbf{h}\mathbf{o}\mathbf{r}\mathbf{e}\:\mathbf{u}\mathbf{n}\mathbf{i}\mathbf{t}\mathbf{s}\:\left(\mathbf{\%}\right)=\left[\frac{\text{A}\text{r}-\text{A}\text{s}}{\text{A}\text{r}}\right]\times\:100$$

Where Ar and As are the reference (CAS reagent) and sample absorbance at 630 nm and, the assay was done in a triplet for each assay.

#### Chemical characterization of *S. mundulus* siderophore

The quantitative assay nominated *S*. *mundulus* for its ability to produce a higher siderophore unit compared to other test isolates.

Variation in siderophore type is attributed to their functional chemical groups that can induce color change used for applying specific assays of type differentiation, upon which the following tests were performed. FeCl_3_ test [34] distinguishes between catecholate and hydroxamate-type siderophores whereas Arnow’s assay [35] and Csaky’s assay [36] were further used to confirm the results.

**FeCl**_**3**_**test** was done by mixing 2 mL of freshly made 2% FeCl₃ solution with culture supernatant (1 mL). The presence of hydroxamate-type siderophores is suggested by the production of an orange color with a distinct peak at 420–450 nm. Catecholate siderophores produce a wine color with a distinct peak at 495 nm [34].

**Arnow’s test** was carried out by combining 0.5 M HCl (1mL) with culture supernatant (1 mL). Nitrate molybdate reagent (1 mL) (prepared by dissolving 10 g of sodium nitrite and 10 g of sodium molybdate in 100 mL of distilled water) then added to 10 M NaOH (1 mL). The reaction mixture was kept at room temperature for 5 min. The formation of yellow coloration indicates the presence of catecholate-type siderophores [35].

**Csaky’s assay** was based on the bounding of hydroxamates that are hydrolyzed to hydroxylamine which then oxidized to nitrite that was detected by the addition of N-naphthyl ethylenediamine and sulfanilamide [36]. In this assay, the culture filtrate (1 mL) was hydrolyzed in 6 N H₂SO₄ (1 mL) for 6 h in a boiling water bath. A 3 mL of 35% sodium acetate solution was added for the pH neutralization after hydrolysis. Once cooled, acid-iodine solution (1 mL) (1% sulfanilic acid in 30% acetic acid mixed with 0.5 mL of 1.3% iodine in 30% acetic acid) was added. After 5 min at room temperature, 1 mL of 2% tri-sodium arsenite (Na₃AsO₂) was added to eliminate any remaining iodine. After that, 1 mL of 0.3% α-naphthylamine in 30% acetic acid was added and left to react for 20 min. The pink color suggested the presence of hydroxamate siderophores [37, 38].

### Optimization of growth variables for maximizing siderophore production

Various parameters including iron and NaNO_3_ concentrations and culture pH were investigated by attaining a single-factor strategy for enhancing *S. mundulus* siderophore production. The effect of different Fe concentrations was studied at the range of 1 × 10^− 5^ to 10 µM with logarithmic intervals related to the findings of Deshmukh and Puranik [28]. The pH value effect on *S. mundulus* growth and siderophore production was studied in the pH range (5–10), while the effect of different NaNO_3_ concentrations was studied within the range of 0.5–3 g L^− 1^. After inoculation with 20 mL 5-day-old *S. mundulus*, cultures were grown on iron-deficient BG11 medium under the previously mentioned growth conditions for 20 days. Then after, culture supernatants were used in siderophore yield assessment via CAS assay, and the collected biomass was dried at 65 °C.

### Experimental design for optimization of siderophore production in *S. mundulus* using the central composite design (CCD)

Response Surface Methodology (RSM) was used to investigate the interactions among key parameters affecting siderophore production. The Central Composite Design (CCD) was applied to optimize these variables and responses [39]. Based on previous one-factor experimental results, three independent variables were selected: X1 (Fe concentration - µM), X2 (pH level), and X3 (NaNO₃ concentration - g L⁻¹). Two dependent variables, dry weight (mg dry biomass L⁻¹) and siderophore units (SU%), were used to design the experiment with CCD, employing Minitab^®^ software. The experiment incorporated three levels for each factor (− 1, 0, + 1), as detailed in Table 1.

**Table 1:** Coded and actual values of the experimental variables used for the CCD matrix

Variable		Levels		
	-1		0	1
Fe concentration (µM)	0.001		0.0055	0.01
Initial pH level	6		7	8
NaNO_3_ concentration (g L^− 1^)	1		1.5	2

A matrix of 20 runs was created, with each trial carried out in duplicate. Each run was inoculated with 20 mL of *S. mundulus* and cultured for 20 days using the previously specified conditions. At the conclusion of the experiment, dry weight biomass and siderophore production were determined. Furthermore, the CCD experimental data were analyzed with the response surface regression method and fitted to a second-order polynomial equation [40].

$$\:Y=\beta\:0+\:\sum\:\beta\:iXi+\sum\:\:\beta\:iiXii+\sum\:\beta\:ijXij$$

Where the predicted response was presented by *Y*, the intercept was denoted by $\:{\beta\:}_{i}$, the linear coefficient for the variable ($\:{X}_{i}$) was presented by ($\:{\beta\:}_{i}$), the nonlinear quadratic coefficient for the variable’s squared term ($\:{X}_{ii}$) was displayed by ($\:{\beta\:}_{ii}$), the polynomial coefficient ($\:{\beta\:}_{ij}$) was used for the interaction between variables ($\:{X}_{ij}$).

### Siderophore extraction

*S. mundulus* was cultured on the optimized medium under the previously mentioned environmental conditions and the cell-free supernatant was used for siderophore extraction following the chloroform-phenol-ether-water protocol [41, 42]. To one liter of the culture supernatant, 0.5 gm of FeCl_3_ was combined resulting in the formation of an orange color to observe the siderophore and keep it stable during the extraction process. About 0.3 NaCl g L^− 1^ was added to remove culture impurities as protein particles [43], then centrifugated at 4193 × *g* for 20 min. The aqueous supernatant was extracted with chloroform-phenol (1:1 v /wt.) in a one-liter separating funnel by which the lower organic phase containing siderophores was taken. The extract was then diluted by adding twice the volume of diethyl ether and half the volume of dist. H_2_O. At this step, siderophores were transferred to the aqueous phase by H_2_O, and the traces of phenol in the aqueous phase were removed by diethyl ether. Two volumes of 3% (w/v) 8-hydroxyquinoline/chloroform [44] were added and kept at room temperature to confirm the complexation between iron and 8-hydroxyquinoline. To remove any excess of 8-hydroxyquinoline the aqueous phase was treated with chloroform repeatedly until clearness. Finally, the product was freeze-dried and kept at 4 °C till use [44, 45].

### Characterization of the isolated siderophores

#### Fourier-Transform Infra-Red (FT-IR) analysis

The dried siderophore sample was mixed with potassium bromide and analyzed with FT-IR spectrophotometry (Thermo Fisher Nicolet iS10, USA) to identify the chemical functional groups. Spectra were estimated over the range of 4000 to 500 cm⁻¹ [46].

#### Proton NMR analysis

The ^1^H NMR spectra of the isolated siderophore were obtained using a BRUKER 500-MHz instrument coupled with a triplet resonance probe and triple-axis gradients. The sample was diluted in 0.75 mL of deuterated dimethyl sulfoxide (DMSO) and analyzed using the ECA 500 II NMR equipment (JEOL, Japan) at Mansoura University’s Faculty of Science. The signal solvent served as the internal standard for nuclear magnetic resonance studies [47].

#### Enhancement of *Zea mays* growth using the extracted siderophore

A homogeneous and pure batch of *Zea mays* seeds was obtained from the Ministry of Agriculture’s Field Crop Institute, Agriculture Research Centre in Giza, Egypt. These seeds were used in the experiment to determine the effect of the extracted siderophores on growth in iron-deficient conditions.

*Zea mays* seeds’ surface was sterilized with 1% sodium hypochlorite solution for two minutes. To eliminate any chlorine residue, the seeds were rinsed with distilled water. Germination bioassays were carried out by the previously established protocol [48]. First, the seeds were steeped in distilled, sterilized water for 12 h. The seeds were germinated in 110 mm Petri plates with wet Whatman filter paper No. 3 for up to four days at 25 ± 1 °C in the dark. Afterward, the four-day-old seedlings were transferred to the different Hogland nutrient solutions (250 mL Glass jars) covered with aluminum foil with continuous aeration [49]. The jars were divided into three groups. The first treatment (T_0_) contained full Hoagland solution (full iron concentration = 64 mM) serving as a positive control, the second treatment (T_1_) contained Hoagland solution under iron limiting conditions where ferric EDTA was omitted, and the third one (T_2_) contained iron-limited Hoagland solution in addition to the purified siderophore (45 mg L^− l^). All jars were incubated at 25 ± 1 ºC with controlled light conditions (16 h light and 8 h dark intervals) for 10 days. The solutions were renewed each with its fresh nutrient solution every 3 days. Polystyrene net sheets were used for holding the free-floating roots. After 14 days, the growth physical parameters were estimated in all treatments, and photosynthetic pigments (chlorophyll a, chlorophyll b, and carotenoids) were assessed according to the protocol of Metzener et al. [50]. After drying the seedlings at 80 °C, the total carbohydrate content was measured using the phenol–sulfuric acid method with glucose as a standard [51], and total protein content was estimated according to the protocol of Lowry et al. [52]. using crystalline bovine serum albumin as a standard. To analyze the elemental composition of the treated seedlings, Energy Dispersive Spectroscopy (EDS) was performed using an energy-dispersive X-ray spectrometer (Oxford X-Max 20) [53].

### Statistical analysis

Statistical analyses were conducted to evaluate treatment responses in the experimental data using various methods. One-way ANOVA was used to assess significant differences between groups, followed by post-hoc comparisons with Tukey’s Honest Significant Difference (HSD) and Fisher’s Least Significant Difference (LSD) tests at a 95% confidence level. Student’s t-test was used for paired group comparisons analysis. For optimization studies, regression analysis of three factors influencing siderophore production was conducted, followed by a Response Surface Methodology (RSM) employing a 20-run Central Composite Design (CCD) model. According to the hypersensitivity of the technique, a confidence level of 90% was used for RSM. The model analysis included an estimation of effects, ANOVA, and a prediction profiler to identify optimal conditions, which were subsequently validated experimentally. All statistical tests, tabulation, modeling, and visualization were performed using Minitab software (version 22) and JMP software (version 17.1).

## Results

### Growth responses and siderophore production of the investigated cyanobacteria grown under iron limitation

**Fig. 1:**
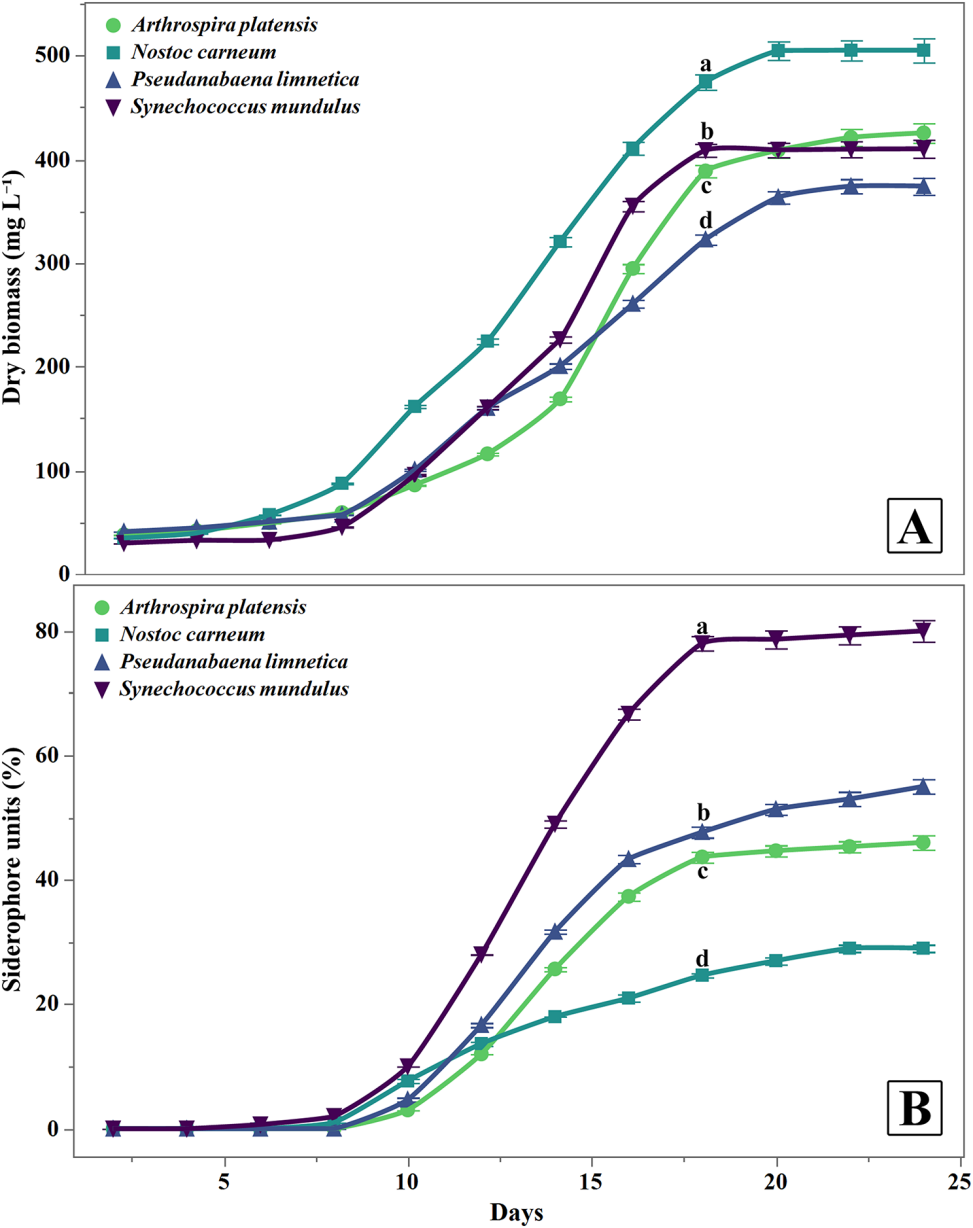
(**A**) Growth responses (mg dry biomass. L^− 1^) and (**B**) Siderophore units (%) of *S. mundulus*, *P. limnetica*, *A. platensis* and *N. carneum* grown under iron limitation for 24 days incubation period. Data are presented as means ± SD of three replications. Different letters indicate significant differences at *p* ≤ 0.05

The growth responses (mg dry biomass L^− 1^) and siderophore units (%) of *S. mundulus*, *P. limnetica*, *A. platensis* and *N. carneum* grown under iron limitation are illustrated in Fig. 1. Generally, growth was increased progressively throughout the incubation period for all the cyanobacterial cultures. The maximum growth was demonstrated on day 22 for *N. carneum*, *A. platensis*, *P. limnetica*, and *S. mundulus* recording 505 ± 17, 421 ± 14, 409.67 ± 13.01 and 374 ± 12 mg L^− 1^, respectively (Fig. 1A). As to siderophore biosynthesis, the maximum siderophore units were achieved by all the tested isolates after 18 days of growth. Whereas the maximum significant siderophore production was observed for *S. mundulus* (78 ± 2%), while the minimum was observed for *N. carneum* (24.67 ± 0.58%). Accordingly, *S. mundulus* was selected for further investigations in this study (Fig. 1B).

### Identification of *S. mundulus* siderophore

**Fig. 2:**
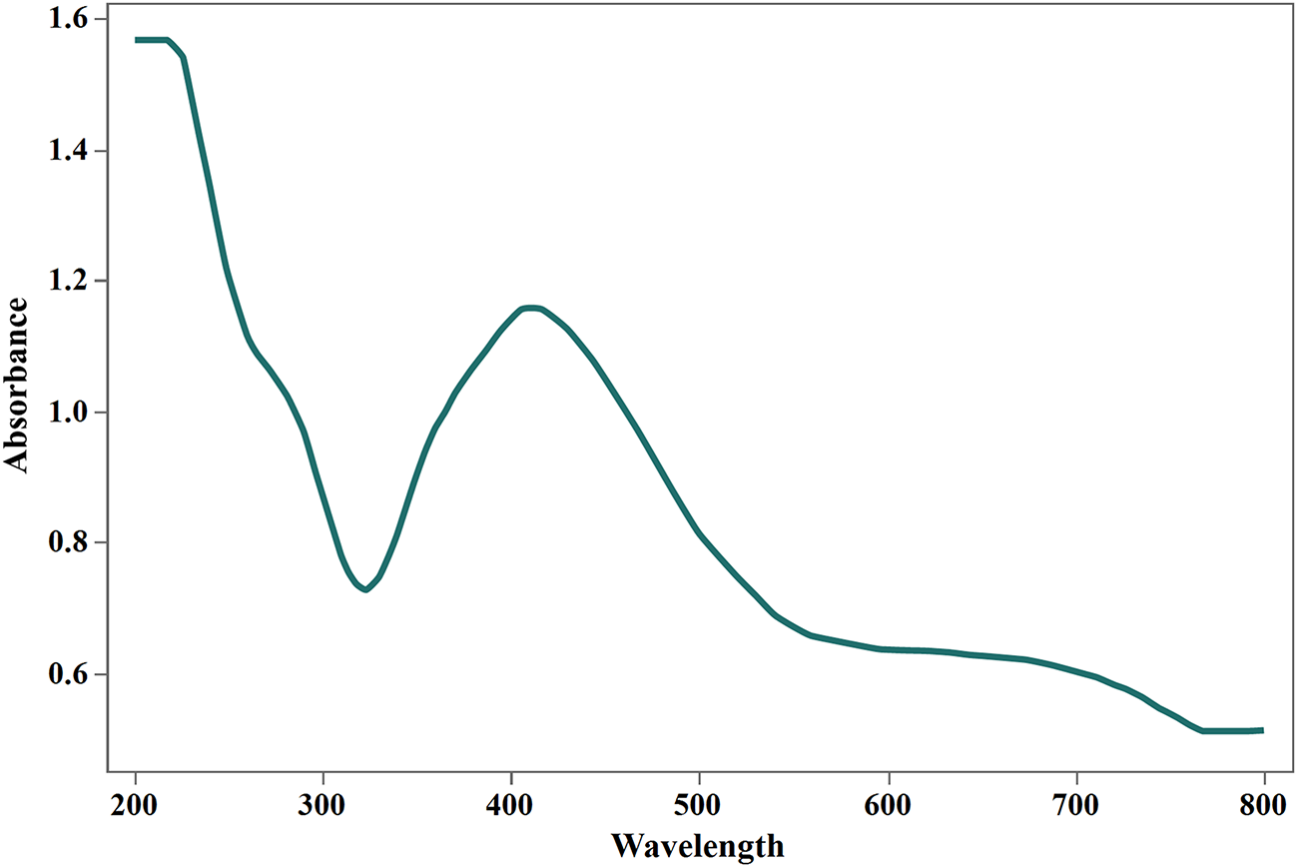
UV-visible absorption spectra of the FeCl_3_ assay reaction indicating presence of hydroxamate siderophore

Regarding the FeCl_3_ test, the culture supernatant developed an orange color with a distinct peak at 424 nm when applied with FeCl_3_ solution in a ratio of 1:2 (Fig. 2), confirming the hydroxamate nature of the produced siderophore. However, using culture filtrate: FeCl_3_ at 1:1 exhibited no wine color, excluding catecholate siderophore. Furthermore, the negative result of the Arnowʼs test and the pink color developed by the Csaky test excluded the hypothesis of the catecholate nature of the obtained siderophores. Consequently, the produced siderophore was found to be of hydroxamate nature.

### Growth conditions optimization via single factor experimental analysis strategy

**Fig. 3:**
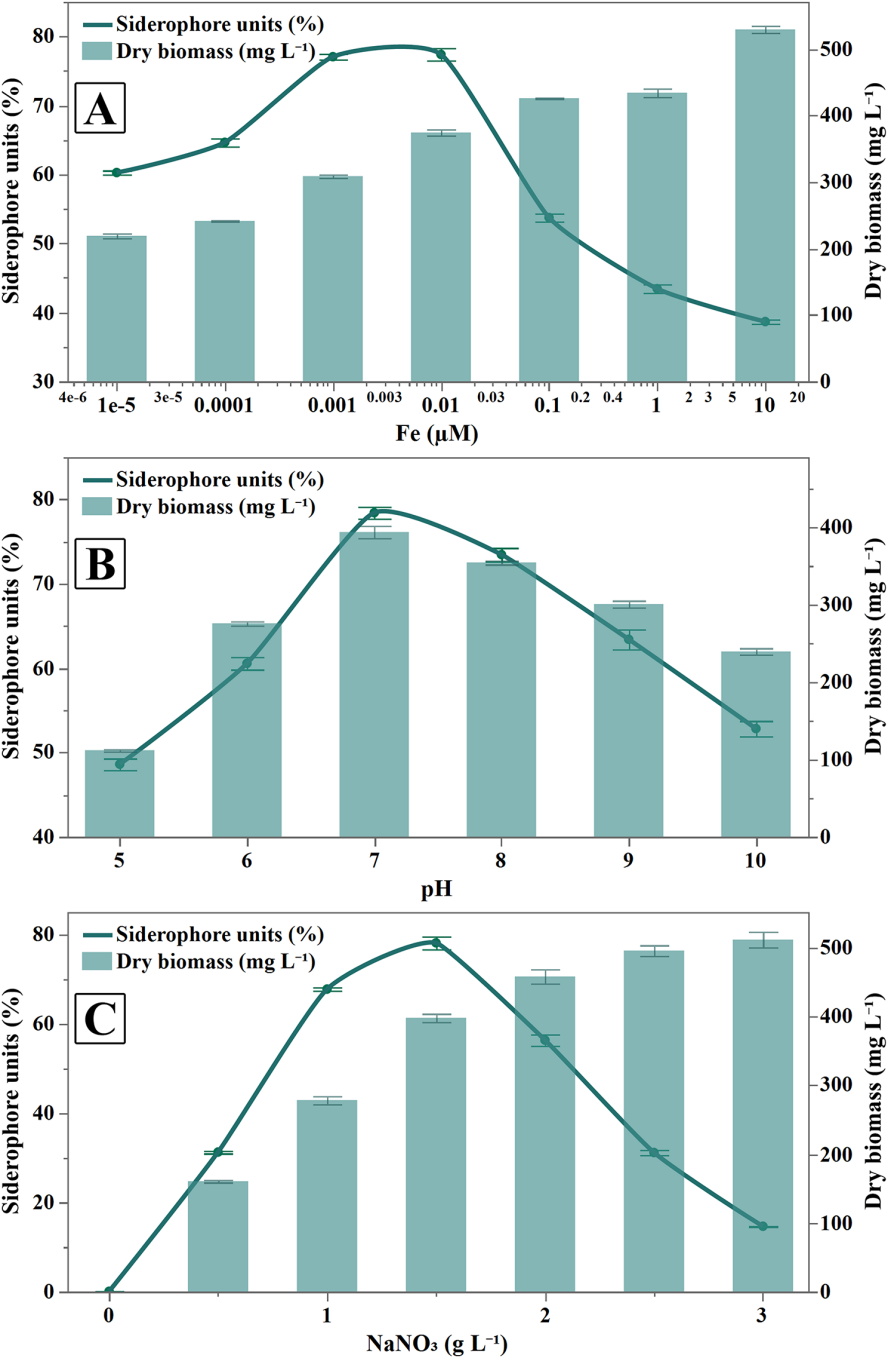
Effect of different culture conditions on siderophore units (%) and growth (g dry biomass L^− 1^) by *S. mundulus.* **A**) Effect of iron conc. (1 × 10^− 5^ to 10 µM with logarithmic interval), **B**) Effect of pH and **C**) Effect of NaNO_3_ concentrations. Data are presented as means ± SD of three replications and the error bar represents ± standard error

The variation in biomass yield (mg dry biomass L^− 1^) and siderophore units (%) in response to different pH, NaNO_3,_ and Fe concentrations are illustrated in (Fig. 3).

Figure 3A illustrates how Fe³⁺ concentrations affect siderophore synthesis and dry weight in *S. mundulus.* The results indicated that the optimal Fe³⁺ concentration for siderophore synthesis was 0.01 µM, yielding 77.39 ± 1.56% SU, and for growth, it was also 0.01 µM, yielding 374.67 ± 8.02 mg dry biomass L⁻¹. As the concentration of Fe³⁺ increased, siderophore synthesis decreased significantly.

The maximum amount of siderophore (78.42 ± 1.2%) was obtained at pH 7 as illustrated in Fig. 3B. Whereas the pH 5 suppressed *S. mundulus* siderophore production (48.62 ± 1.18%). A gradual decrease in siderophore production with increasing alkalinity was observed. For growth, the maximum biomass production (394 ± 14 mg dry biomass L⁻¹) was achieved at a neutral pH of 7, while both acidic and alkaline conditions significantly reduced biomass production. Consequently, the pH was maintained at 7 to optimize the production of siderophore in subsequent experiments.

The optimal NaNO₃ concentration for maximal siderophore production in *S. mundulus* was investigated. The highest siderophore production, measuring 78.12 ± 2.44%, was recorded in 1.5 g L⁻¹ NaNO₃ containing medium, as shown in Fig. 3C. While the production of siderophore was suppressed with increasing NaNO_3_ to 3 g L^− 1^ reaching (14.58 ± 0.15%). On the contrary, *S. mundulus* growth was increased almost more than threefold by increasing NaNO_3_ concentration in the medium from 0.5 to 3 g L^− 1^. However, in a medium containing 1.5 g L^**− 1**^ NaNO_3_, *S. mundulus* maintained 398.33 ± 10.26 mg dry biomass L^− 1^. Thus, this NaNO₃ concentration was selected for optimal siderophore production in subsequent experiments.

### Siderophore production condition optimization using response surface methodology

**Fig. 4:**
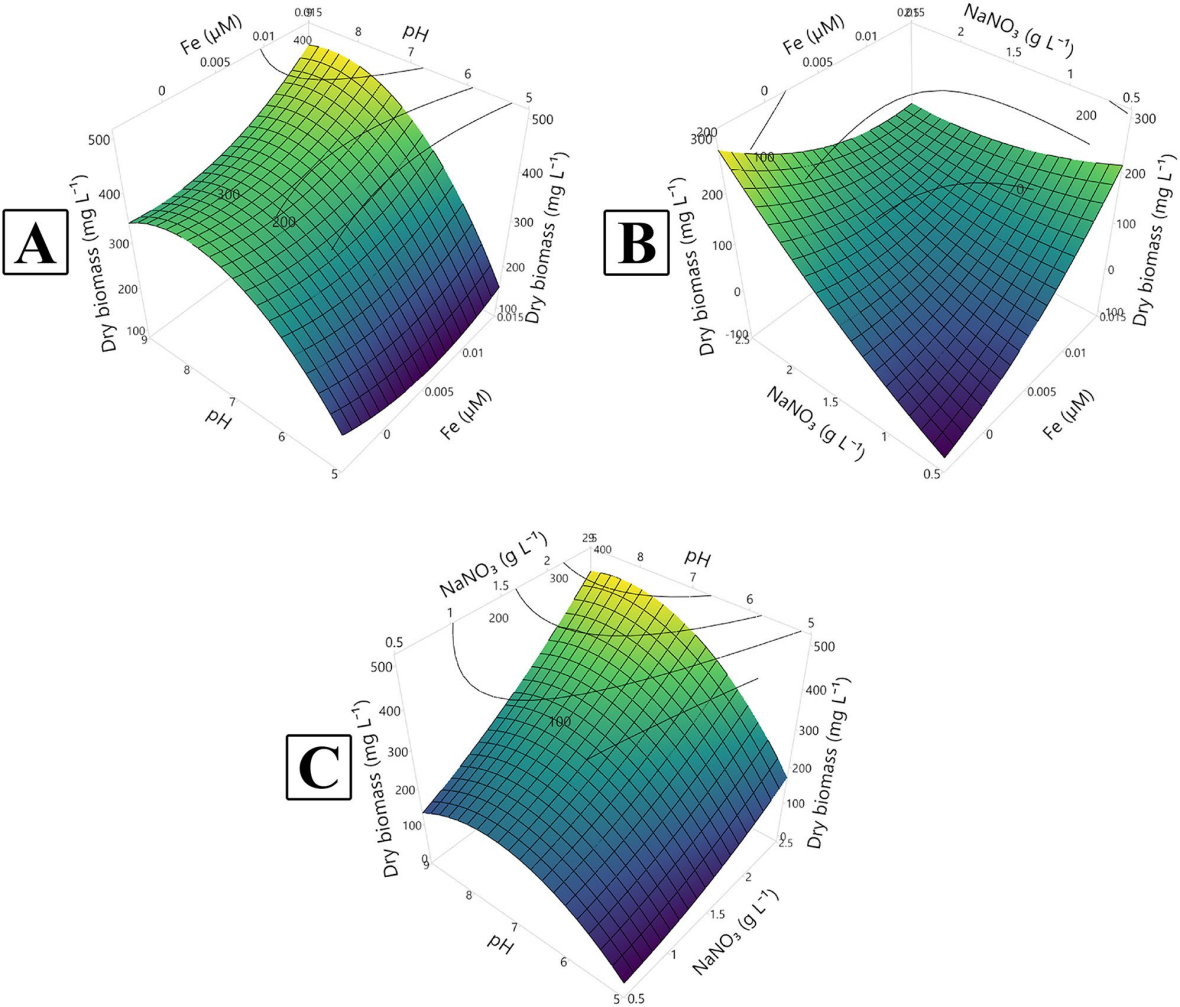
Three-dimensional response surface plot (**A**–**C**) showing the interactions between the tested variables (iron conc., pH and NaNO_3_ conc.) for the maximum production of biomass (mg dry biomass L^− 1^)

**Fig. 5:**
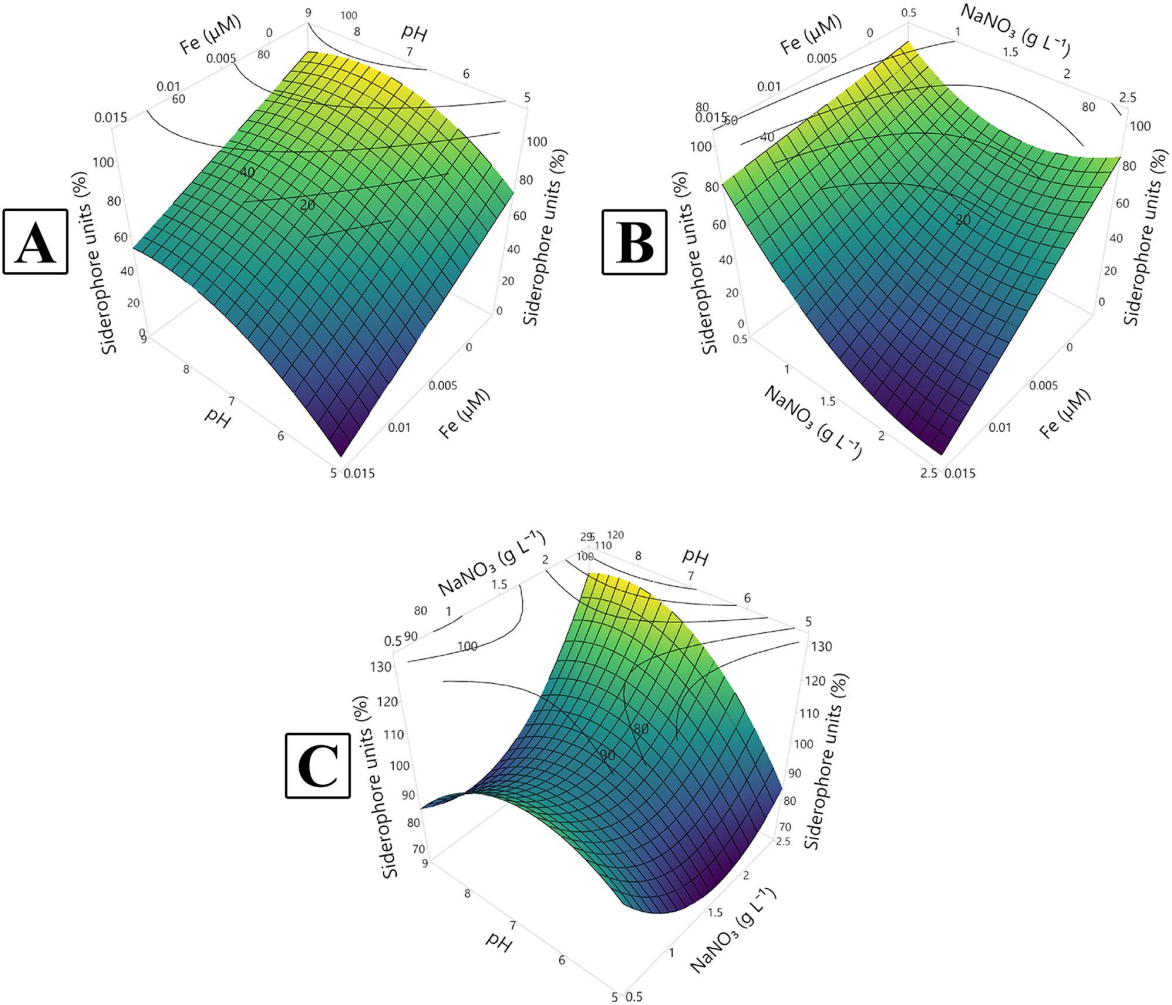
Three-dimensional response surface plot (**A**–**C**) showing the interactions between the tested variables (iron conc., pH and NaNO_3_ conc.) for the siderophore maximum production

A Central Composite Design (CCD) matrix was employed to identify the optimal pH, NaNO₃, and Fe³⁺ concentrations for maximizing siderophore production (% SU) and biomass production (mg dry biomass L⁻¹). The resulting matrix and the design responses are detailed in Tables 2 and 3 and illustrated in Figs. 4 and 5 which demonstrates a matrix of twenty trials including 8 factorial, 6 axial, and 6 central points.

**Table 2:** Central Composite Design (CCD) matrix of the three studied factors (iron conc., pH and NaNO_3_ conc.) With the experimental values for siderophore units (%) and biomass (g dry biomass L^− 1^) production

Run	Fe (µM) (X_1_)	NaNO₃ (g L⁻¹) (X_2_)	pH (X_3_)	% SU	Dry Wt. (mg L⁻¹)
1	-0.00207	1.5	7	77.55	165.75
2	0.001	1	6	92.7	185.86
3	0.001	1	8	93.58	249.06
4	0.001	2	6	83.77	279.91
5	0.001	2	8	92.83	392.69
6	0.0055	0.65	7	86.52	106.05
7	0.0055	1.5	5.3	40.42	94.69
8	0.0055	1.5	7	73.92	283.7
9	0.0055	1.5	7	73.92	283.7
10	0.0055	1.5	7	73.92	283.7
11	0.0055	1.5	7	73.92	283.7
12	0.0055	1.5	7	73.92	283.7
13	0.0055	1.5	7	73.92	283.7
14	0.0055	1.5	8.7	71.18	246.41
15	0.0055	2.34	7	75.84	420.7
16	0.01	1	6	77.15	253.99
17	0.01	1	8	77.33	354.61
18	0.01	2	6	47.76	255
19	0.01	2	8	66.53	400.16
20	0.013068	1.5	7	57.22	379.46

**Table 3:** Analysis of variance (ANOVA) of the CCD model for siderophore production and dry biomass in response to iron conc., pH and NaNO_3_ conc

Source	% SU			Dry biomass (mg L^− 1^)					
	Adj SS^a^	Adj MS^b^	F-Value^c^		*P*-Value^d^	Adj SS	Adj MS	F-Value	*P*-Value
Model	2883.53	320.39	4.9		0.01	120,957	13439.7	4.37	0.015
Linear	2018.19	672.73	10.29		0.002	101,471	33823.5	10.99	0.002
Fe (µM)	1205.34	1205.34	18.43		0.002	19,470	19470.2	6.33	0.031
NaNO₃ (g L⁻¹)	336.91	336.91	5.15		0.047	48,448	48447.9	15.74	0.003
pH	475.94	475.94	7.28		0.022	33,553	33552.6	10.9	0.008
Square	649.25	216.42	3.31		0.066	13,204	4401.4	1.43	0.291
Fe (µM)*Fe (µ)	0.04	0.04	0		0.982	1313	1313.1	0.43	0.528
NaNO₃ (g L⁻¹)*NaNO₃ (g L⁻¹)	349.89	349.89	5.35		0.043	569	568.8	0.18	0.676
pH*pH	235.89	235.89	3.61		0.087	10,148	10148.1	3.3	0.099
2-Way Interaction	216.08	72.03	1.1		0.393	6282	2094.1	0.68	0.584
Fe (µM)*NaNO₃ (g L⁻¹)	116.36	116.36	1.78		0.212	4566	4565.9	1.48	0.251
Fe (µM)*pH	10.15	10.15	0.16		0.702	609	609	0.2	0.666
NaNO₃ (g L⁻¹)*pH	89.58	89.58	1.37		0.269	1107	1107.3	0.36	0.562
Error	654.07	65.41				30,775	3077.5		
Lack-of-Fit	654.07	130.81	*		*	30,775	6155.1	*	*
Pure Error	0	0				0	0		
Total	3537.59					151,732			

^a^ Sum squares

^b^ Mean squares

^c^ Fishers’s function

^d^ P level of significance

Data in Table 2 revealed that the maximum siderophore production reached 93.58%SU in response to run 3 while the maximum dry biomass was 420.7 mg dry wt. L^− 1^ in response to the run 15. Significance was determined at a 90% confidence level, with p-values < 0.1 showing reciprocal interactions between variables. Analysis using multi-way ANOVA (Table 3) revealed that the model was significant for both siderophore production and dry biomass, with p-values of 0.01 and 0.015, respectively. For siderophore production, the linear coefficient terms (X_1_, X_2_, X_3_) and the terms of quadratic coefficient (X₁², X₂², X₃²) showed significant responses, while the terms of interaction coefficient (X_1_ × _2_, X_1_ × _3_, X_2_ × _3_) were not significant. The most significant variables affecting the siderophore production were initial Fe^+ 3^ (µM), NaNO_**3**_ (g L^− 1^) concentrations and pH levels with a p-value of 0.002, 0.047 and 0.022, respectively as illustrated in Table 3. However, for biomass production, only the linear coefficient terms (X_**1**_, X_**2**_, X_**3**_) demonstrated significant responses. The most significant variables affecting biomass production were initial Fe^+ 3^ oncentration (µM), NaNO_**3**_ (g L^− 1^) concentrations and pH level with p-values of 0.031, 0.003 and 0.008, respectively as illustrated in Table 3. The results indicated an R² value of 81.51% for siderophore production and 79.72% for biomass production, which aligns with the adjusted computed coefficient R² values of 64.87% and 61.46%, respectively. These values reflect the model’s high significance.

**Table 4:** The actual and predicted values for siderophore units and dry biomass production collectively induced by the optimized growth conditions

Variables			Actual values		Predicted values	
Fe^3+^ (µM)	NaNO_3_ (g L^− 1^)	pH	Siderophore unit (%)	Dry biomass (mg L^− 1^)	Siderophore unit (%)	Dry biomass (mg L^− 1^)
0.001	2	8	91.84 ± 0.72	387.11 ± 4.56	90.86	369.23

Equations 1 and 2 demonstrate second-order polynomial models for predicting biomass dry weight and siderophore production as a function of the experimental variable. The model was validated practically.

For biomass dry weight (Eq. 1):

1 $$\begin{aligned}Dry{\text{ }}biomass{\text{ }}\left({mg{\text{ }}{L^{ - 1}}} \right){\text{ }} = &{\text{ }} - 1286{\text{ }} + {\text{ }}5560{\text{ }}Fe{\text{ }}\left({\mu M} \right){\text{ }} - {\text{ }}63{\text{ }}NaNO{\text{ }}\left({g{\text{ }}{L^{ - 1}}} \right){\text{ }} \\&+ {\text{ }}375{\text{ }}pH{\text{ }} + {\text{ }}471386{\text{ }}Fe{\text{ }}\left({\mu M} \right)*Fe{\text{ }}\left({\mu M} \right){\text{ }} \\&+ {\text{ }}25.1{\text{ }}NaNO{\text{ }}\left({g{\text{ }}{L^{ - 1}}} \right)*NaNO{\text{ }}\left({g{\text{ }}{L^{ - 1}}} \right){\text{ }} \\&- {\text{ }}26.5{\text{ }}pH*pH - {\text{ }}10618{\text{ }}Fe{\text{ }}\left({\mu M} \right)*NaNO{\text{ }}\left({g{\text{ }}{L^{ - 1}}} \right){\text{ }} \\&+ {\text{ }}1939{\text{ }}Fe{\text{ }}\left({\mu M} \right)*pH{\text{ }} + {\text{ }}23.5{\text{ }}NaNO{\text{ }}\left({g{\text{ }}{L^{ - 1}}} \right)*pH\end{aligned}$$

For Siderophore production (Eq. 2):

2 $$\begin{aligned}\% {\text{ }}SU{\text{ }} =& {\text{ }} - 29{\text{ }} - {\text{ }}1324{\text{ }}Fe{\text{ }}\left({\mu M} \right){\text{ }} - {\text{ }}106.6{\text{ }}NaNO{\text{ }}\left({g{\text{ }}{L^{ - 1}}} \right){\text{ }}\\& + {\text{ }}51.1{\text{ }}pH{\text{ }} + {\text{ }}2475{\text{ }}Fe{\text{ }}\left({\mu M} \right)*Fe{\text{ }}\left({\mu M} \right){\text{ }} \\&+ {\text{ }}19.71{\text{ }}NaNO{\text{ }}\left({g{\text{ }}{L^{ - 1}}} \right)*NaNO{\text{ }}\left({g{\text{ }}{L^{ - 1}}} \right){\text{ }} \\&- {\text{ }}4.05{\text{ }}pH*pH{\text{ }} - {\text{ }}1695{\text{ }}Fe{\text{ }}\left({\mu M} \right)*NaNO{\text{ }}\left({g{\text{ }}{L^{ - 1}}} \right){\text{ }} \\&+ {\text{ }}250{\text{ }}Fe{\text{ }}\left({\mu M} \right)*pH{\text{ }} + {\text{ }}6.69{\text{ }}NaNO{\text{ }}\left({g{\text{ }}{L^{ - 1}}} \right)*pH\end{aligned}$$

The response surface plots (Figs. 4 and 5) depict the interactions between the variables investigated to optimize siderophore and biomass production. The lighter coloration on the plots indicates the direction of the optimal conditions for each response.

For maximizing biomass production to more than 400 mg dry biomass L^− 1^, the optimum Fe^**+** 3^ concentration was found to be 0.005 µM whereas the optimum initial pH was 7–9 (Fig. 4A). Whereas the optimum NaNO_**3**_ concentration was more than 2 g L^**− 1**^ and pH value 7–9 (Fig. 4B). Regarding the interaction between NaNO_**3**_ concentration (g L^**− 1**^) and Fe^**+ 3**^ concentration (µM), the model predicted that the optimum Fe^**+ 3**^ concentration was less than 0.005 µM and the optimum NaNO_**3**_ concentration was more than 2 g L^− 1^ (Fig. 4C).

Regarding siderophore production, the model predicted that for enhancing siderophore production for 90%, the optimum Fe^**+** 3^ concentration was found to be less than 0.005 µM whereas the optimum initial pH was 7–9 (Fig. 5A). Concerning the interaction between NaNO_**3**_ concentration (g L^**− 1**^) and initial pH (Fig. 5B), the optimum NaNO_**3**_ concentration was more than 2 g L^**− 1**^ and pH value was 7–9. In the interaction between NaNO_**3**_ concentration (g L^**− 1**^) and Fe^**+ 3**^ concentration (µM), the model predicted that the optimum Fe^**+ 3**^ concentration was less than 0.005 µM and the optimum NaNO_**3**_ concentration was more than 2 g L^− 1^ (Fig. 5C).

The model predicted that the maximum values of siderophore (90.86%SU) and biomass production (369.23 mg dry wt. L^− 1^) obtained at NaNO_**3**_ concentration (2 g L^**− 1**^) and Fe^**+ 3**^ concentration (0.001 µM) and pH = 8. The model was validated experimentally, and the actual values were compared to the models’ estimates in Eqs. (1 and 2), and the resulting values are displayed in Table 4.

### Characterization of *S. mundulus* hydroxamate siderophore

**Fig. 6:**
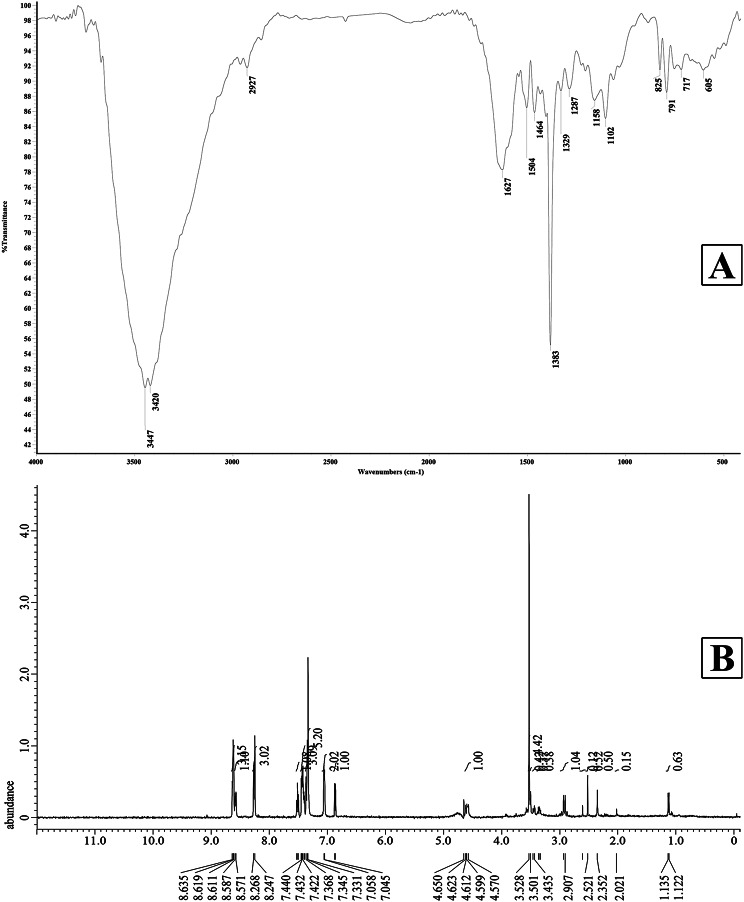
(**A**) FT-IR spectrum, (**B**) H NMR spectrum of the isolated siderophore produced by *S. mundulus*

The FT-IR spectrum of *S. mundulus* siderophore revealed the chemical characteristic functional groups in 4000–400 cm ^− 1^ as illustrated in Fig. 6A and recorded in Table 5 signifying the presence of hydroxamate-type siderophore.

**Table 5:** FT-IR profile of *S. mundulus* siderophore

Wave number range (cm ^− 1^)	Assignments	*S. mundulus* siderophore frequency (wave number cm^− 1^)
3500 − 3200	Aromatic OH group, N-H of the secondary amide	3447, 3420
3300 − 2500	Asymmetric CH_**3**_ stretching	2927
2260 − 1600	C = O stretching	1627
1500 − 1400	C-NH amide II	1504,1464
1360 − 1250	Aromatic C-N stretching	1287
1250 − 1020	Aliphatic C-N stretching	1158,1102
900 − 805	N-O stretching	825

^**1**^H NMR spectrogram of *S.mundulus* hydroxamate siderophore exhibited several peaks (Fig. 6B; Table 6) including peaks ranged 1.122–1.135 ppm indicating = CH group, 2.021–2.907 ppm recognizing CH_2_ = CH and chemical shift at 3.435–3.528 ppm signifying aliphatic OH in addition to CH_2_ (glycine). Also, a peak is identified at the range 4.570–4.650 pointing to C-H (α-D-ornithin), in addition to chemical shifts ranged 7.045–7.368 ppm and 7.422–7.440 ppm specifying the presence of H-5, H-8 aromatic signals (meta benzoxazole, para benzoxazole & oxazole) and Dihydroxy benzoate (DHB), respectively as well as a peak at the range 8.247–8.635 ppm donating the presence of = NH (α-glycine amide) and (α-D-ornithin amide).

**Table 6:** ^1^H NMR assignments (ppm) for the purified siderophore

^1^H atom (moiety)	Chemical shift (ppm)
=CH	1.122–1.135
CH_2_ = CH	2.021–2.907
aliphatic OH CH_2_ (glycine)	3.435–3.528
C-H (α-D-ornithin)	4.570–4.650
H-5, H-8 aromatic signals (meta benzoxazole, para benzoxazole & oxazole) Dihydroxy benzoate (DHB)	7.045–7.368 7.422–7.440
=NH (α-glycine amide) (α-D-ornithin amide)	8.247–8.635

### *Zea mays* growth responses to siderophore supplementation under iron deficiency

The growth physical parameters of maize seedlings are listed in Table 7. Significant increments were observed in shoot and root lengths grown in response to free-iron Hoagland solution supplemented with siderophore (T_2_) reaching 28.8% and 53.84%, respectively compared to the control. Seedling growth in free-iron Hoagland solution (T_1_) induced significant decrements reached 22.36% and 23.07% in shoot height and root length, respectively. *Zea* seeds are grown in siderophore siderophore-supplemented medium (T_2_) significantly enhanced seedling growth expressed as dry biomass documenting a 65.39% increase. In the case of T_1_ seedling growth, an opposite trend of response was found, resulting in a considerable decrease in dry biomass, with the degree of response recorded as 23.78%. Seedlings growth under the T_2_ condition exhibited the maximum significant increase in leaf area reaching 41.83% while, a significant decrease of 35.45% in leaf area was recorded in response to T_1_. The non-significant difference was recorded in maize leaf number in response to T_1_ and T_2_ compared with the control (T_0_).

**Table 7:** Effect of *S. mundulus* siderophore supplimentation on growth criterea of 18-day-old *Zea mays* seedlings. Data represents mean ± SD, *n* = 10. T_1_: Hoagland solution in absence of iron, and T_2_: iron limited Hoagland solution in addition to the purified siderophore. Different letters indicate significant differences at *P* ≤ 0.05

Treatment	Seedling dry weight (g)	Shoot length (cm)	Root length (cm)	Leaf area (cm²)	No. of leaves
T₀	1.257 ± 0.018 ^b^	18.033 ± 0.862 ^b^	13 ± 1.732 ^b^	10.11 ± 1.5 ^ab^	3 ± 0 ^a^
T₁	0.958 ± 0.016 ^c^	14 ± 0.5 ^c^	10 ± 2 ^b^	6.525 ± 1.405 ^b^	2 ± 1 ^a^
T₂	2.079 ± 0.061 ^a^	23 ± 2 ^a^	20 ± 1 ^a^	14.34 ± 2.349 ^a^	3 ± 1 ^a^
Prob > F	< 0.0001	0.0004	0.0008	0.0054	0.2963
LSD 0.05	**0.08**	**2.58**	**3.26**	**3.6**	**1.63**

**Fig. 7:**
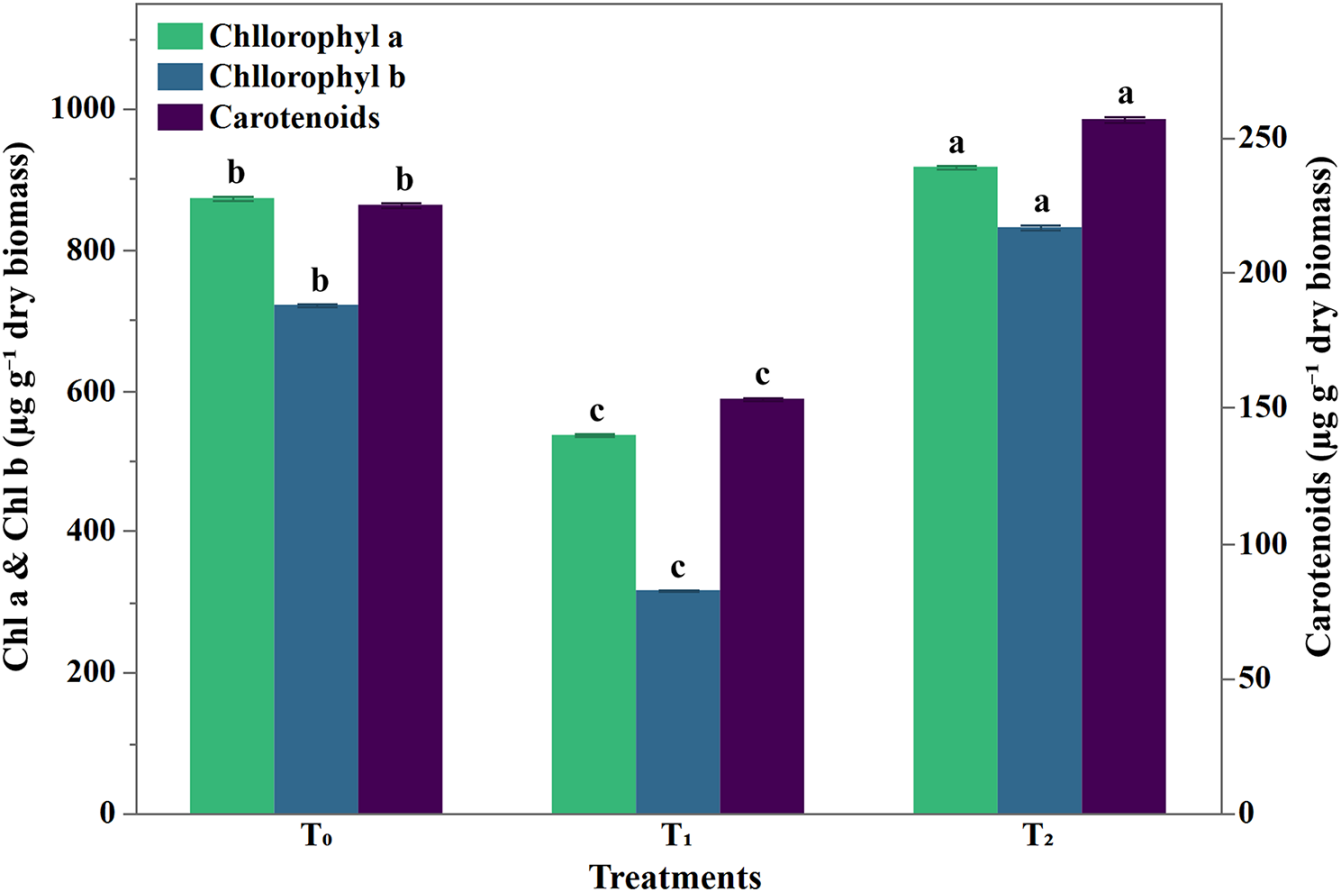
Effect of *S. mundulus* siderophore supplementation on photosynthetic pigment concentration (µg g^− 1^ dry wt.) in *Zea mays* plant. Data represents mean ± SD, *n* = 10. Different letters indicate significant differences at *P* ≤ 0.05. T_1_: Hoagland solution in absence of iron, and T_2_: iron-limited Hoagland solution in addition to the purified siderophore

Siderophore supplementation (T_2_) enhanced pigmentation in maize seedlings resulting in remarkable increments of 5.1%, 15.27% and 14.1% in chlorophyll a, chlorophyll b and carotenoids contents, respectively relative to the control (Fig. 7).

**Fig. 8:**
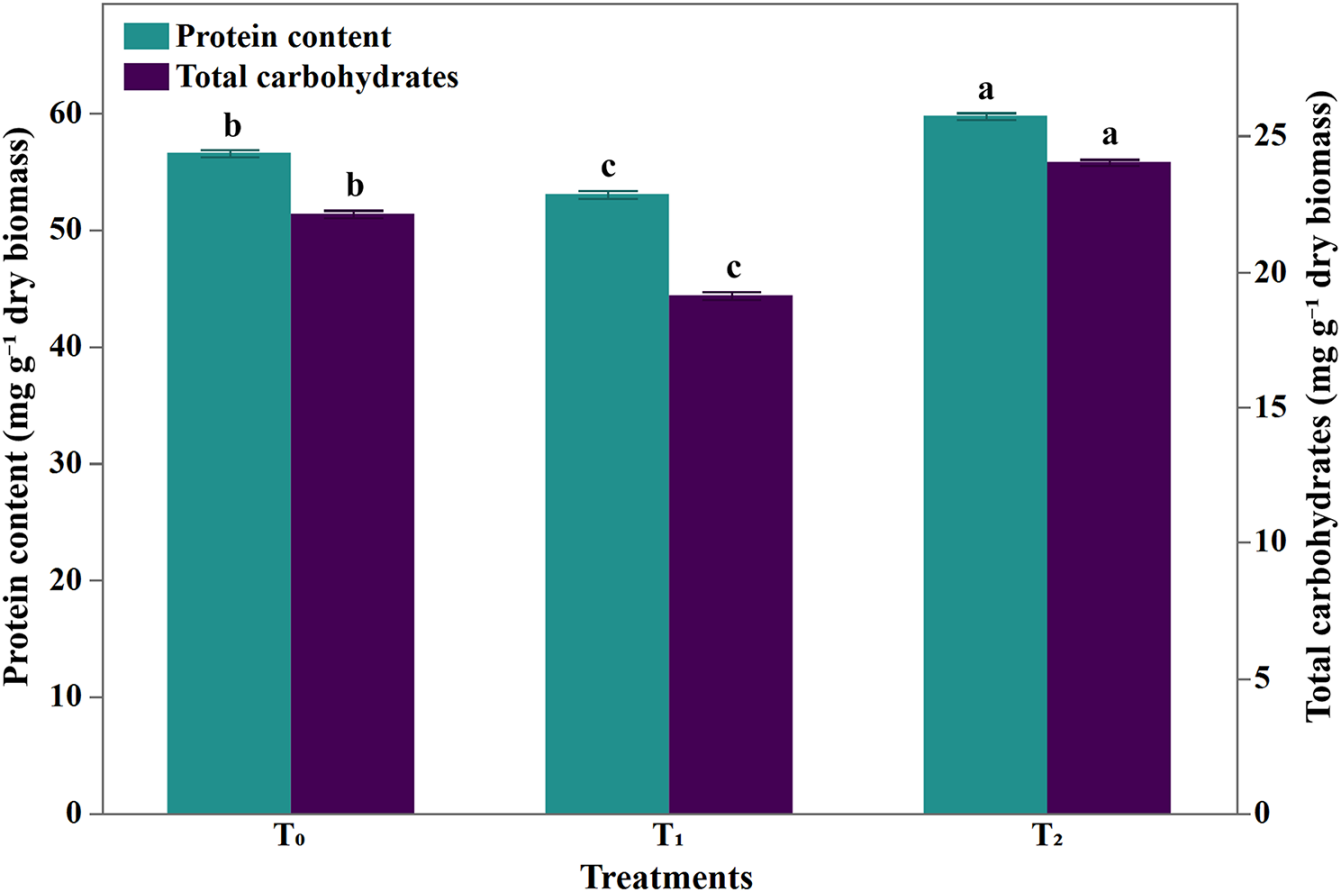
Effect of siderophore on carbohydrate and protein contents (mg g^− 1^ dry wt.) of *Zea mays* growing seeds. Data represents mean ± SD, *n* = 10. Different letters indicate significant differences at *P* ≤ 0.05. T_1_: Hoagland solution in absence of iron, and T_2_: iron-limited Hoagland solution in addition to the purified siderophore

The absence of iron (T_1_) led to a significant decrement of 13.6% in carbohydrate fraction content in the growing maize seedlings as illustrated in Fig. 8. On the contrary, the siderophore-supplemented Hoagland solution significantly (T_2_) stimulated the total carbohydrate content by 8.62%. A significant increase in total soluble protein content (59.71 ± 0.91 mg g^− 1^) of maize seedlings treated with siderophore-supplemented Hoagland solution (T_2_) was detected compared with control (56.53 ± 0.97 mg g^− 1^) (Fig. 8).

### Elementary analysis using Energy Dispersive X-ray spectroscopy (EDX)

Iron content was significantly enhanced by siderophore supplementation relative to control (T_0_) ranging from 0.52% for control to 0.80% weight for iron-free Hoagland media supplemented with siderophore (T_2_) as indicated by elementary analysis (EdX) (Table 8).

**Table 8:** Energy Dispersive X-Ray (EDX) elementary analysis in growing maize seedlings

Element	T₀		T₁		T₂	
	Wt.%	Atomic %	Wt.%	Atomic %	Wt.%	Atomic %
Al	9.31	6.54	-	-	4.2	2.94
Ca	1.76	0.83	5.82	271	3.05	1.44
Cl	1.85	0.99	1.07	0.56	2.24	1.2
Fe	0.52	0.18	0.01	0	0.8	0.27
K	13.4	6.5	12.25	5.84	13.89	6.71
O	70.01	83.04	75.03	87.42	71.91	84.97
P	2.2	1.35	4.13	2.49	2.65	1.62
S	0.95	0.56	1.68	0.98	-	-
Si	-	-	-	-	1.25	0.84

## Discussion

Iron is a crucial trace element for plant growth, where the amount of soluble iron has a significant impact on plant health. Iron deficiency can lead to plant death at the seedling stage, ultimately reducing agricultural productivity [5]. Microorganisms including cyanobacteria have evolved special iron-chelating agents (siderophores) to overcome iron restrictions, which can be employed as a greener and more sustainable technique to stimulate plant growth in iron-free environments [15, 54]. Accordingly, the present work aims to optimize siderophore and biomass production in *S. mundulus* for enhancing *Zea mays* seedling growth in hydroponic-iron limited-based culture.

The results revealed a significant decrease in the biomass yield of the tested isolates under iron limitation. This decrease may be attributed to a reduced growth rate, which could either indicate a decrease in growth requirements or an increase in the cells’ ability to acquire iron. It has been documented that iron deficiency can impact phytoplankton in two distinct ways: reducing the rate of photosynthesis and/or biomass yield [55]. The variation in siderophore production among *S. mundulus*, *P. limnetica*, *A. platensis*, and *N. carneum* can be attributed to both phenotypic and genotypic variance, as well as species-specific factors [20], which aligns with the present findings, which showed that all four cyanobacterial species produced siderophores with varying levels of potential, despite being cultured under similar media and incubation conditions. Siderophores are iron-specific molecules produced in response to low iron availability; thus, elevated iron levels suppress their synthesis. However, a relatively small amount of siderophores is sufficient to fulfill the iron requirements of microorganisms.

The elevated siderophore production observed for the isolates investigated during the late log phase is largely a result of cells depleting their intracellular iron reserves to maintain growth. As intracellular iron diminishes, the regulation of siderophore synthesis is triggered to capture ferric ions from the external environment [26]. It has been reported that the halotolerant *Synechococcus* sp. PCC 7002, exhibited the highest siderophore activity after five days of inoculation [56].

Since cyanobacterial siderophores vary in the functional groups that chelate ferric iron including hydroxamate [57], catecholate [58], carboxylates and alfa-hydroxy carboxylates [59], identifying tests such as the FeCl_3_, Arnow’s, and Csaky confirmed the hydroxamate nature of the siderophore produced by *S. mundulus* herein. The most frequent type of siderophore produced by cyanobacteria is hydroxamate, which consists mostly of C (= O) N-(OH) R [R = an amino acid or its derivative] where two oxygen groups form a bidentate ligand with iron resulting in a hexadentate octahedral complex synthesis [60]. These findings are comparable with those documented by Rashmi et al. [61]. who found opposite results in Arnow’s assay for the extracellular environment, showing the absence of catecholate siderophores in *Synechococcus elongatus* BDU 130,911. Furthermore, the spectroscopic study of *S. mundulus* siderophores showed their hydroxamate origin, generating an orange color, and the produced complex had an absorbance maximum of 424 nm, as previously stated by Howard et al. [62]. Also, Boiteau [63] proved the hydroxymate-type of the siderophores produced from *Synechococcus* sp. PCC 7002.

Regarding *S. mundulus* siderophore yield, culture conditions such as iron concentration, NaNO_3_ (nitrogen source) concentration and pH were adjusted to optimize high siderophore production. Yu et al. [23] and Chakraborty et al. [21] indicated the significance of media pH and nitrogen source (nitrate salts) for enhancing siderophore production in cyanobacteria. The present results indicated a reciprocal correlation between the amount of iron and siderophore yield whereas, the minimum Fe^+ 3^ concentration 0.01 μm induced the maximum production of siderophore. These findings were in harmony with those of Singh and Mishra [26] who found high siderophore production in *Anabaena oryzae* as the initial FeCl_3_ concentration decreased, however higher iron concentrations significantly inhibited siderophore production. On the contrary, Raghuvanshi et al. [25] revealed that increasing iron content to 60 µM inhibited siderophore production in *Anabaena cylindrica*.

Iron solubility and availability to microorganisms are strongly influenced by the pH effect. The present results align with those of Subramanium and Sundaram [64] who reported that pH 7 induced the maximum siderophore production in *Pseudomonas aeruginosa*. Also, Sinha et al. [65] proposed that pH levels close to 8 and 8.2 favored maximum siderophore and growth production in certain bacteria. Siderophores bind Fe³⁺, which is less available in oxygenated, alkaline saltwater.

Present results demonstrated that 1.5 g L^− 1^ sodium nitrate was the optimum for both *S. mundulus* growth and production of siderophore. Kumar et al. [66]. found that at pH 8, both *Pseudomonas fluorescence* and *Pseudomonas putida* produced 60–80% siderophore units from glucose and sucrose and sodium nitrate.

Response Surface Methodology successfully optimized the conditions for siderophore production in *S. mundulus*, achieving actual values of 387.11 mg L⁻¹ for maximum biomass and 91.84% for maximum siderophore production. These values are relatively higher than the predicted values shown in Table 4.

FT-IR analysis was utilized to analyze the characteristic functional chemical groups of the extracted siderophore. Spectral peaks ranging from 4000 to 3400 cm⁻¹ were assigned to -OH and amine functional groups. The FT-IR spectrum prominently displayed strong absorption bands at 3447 and 3420 cm⁻¹, corresponding to the N-H groups of secondary amides and aromatic -OH groups in the siderophores [67]. Murugappan et al. [68] reported a comparable hydroxyl peak at 3357.21 cm⁻¹ and a significant signal at 1042.34 cm⁻¹, signifying the existence of a main aliphatic alcoholic group. Furthermore, the signal at 2950 cm⁻¹ indicated asymmetric CH₃ stretching and saturated alkanes [69] and [67]. The spectrogram confirms the existence of an amide bond in the structure of siderophore. The spectral band at 1627 cm⁻¹ is attributed to amide C = O stretching, indicating bonding between the NH₂ and COOH groups. The signal at 1464 cm⁻¹ indicates -C-H linked with the -CH₂ group and a -N-O structure, suggesting hydroxamate groups [67]. The peak at 1287 cm^− 1^ suggests aromatic C-N stretching [69], whereas the spectral band at 1102 and 1158 cm^–1^ referred to aliphatic C-N stretching [70].

NMR analysis revealed the recognition of the amide group around 8.247–8.635 resonance which signifies the presence of = NH (α-glycine amide) and (α-D-ornithin amide) documenting the existence of a dihydroxamate siderophore-related to the foundations of Carran et al. [71]. Murugappan et al. [72] indicated that *Vibrio harveyi* hydroxamate siderophore demonstrated three triplet peaks at 0.84, 0.91 and 2.18 ppm according to the presence of **CH**_**2**_ -CH_2_, CH_2_ -**CH**_**2**_ and CH_2_ = **CH**, respectively as well as the presence of a double peak at 1.27 ppm revealed **CH**_**2**_ = CH, and three singlet bands localized at 1.35, 1.25 and 3.37 ppm referring to CH_2_ OH, = CH and aliphatic OH. The current spectrum data are consistent with those of Winkelmann et al. [73] and Murugappan et al. [68] who reported that marine *Vibrio* species produced dihydroxamate siderophores (bisucaberin and aerobactin). Also, the present results are in accordance with Ito and Butler [56] who reported the presence of an amid proton resonance at 7.94, a singlet methyl peak at 1.97, and a peak at 1.24 which may have resulted from an alkyl group.

Siderophore-producing microbes synthesize various iron-chelating compounds that can alleviate iron deficiency and enhance physiological and biochemical processes in plants under stressed soil conditions. Therefore, these microbes have potential as biofertilizers for sustainable agriculture and improved crop production [16, 74].

Siderophore supplementation induced significant positive responses on maize seedling growth, morphological criteria, and physiological parameters, grown on limited-iron Hoagland solution in hydroponic system. The present data are in accordance with those of Ghavami et al. [75] who found that siderophore-producing *Micrococcus yunnanensis* and *Stenotrophomonas chelatiphaga* greatly boosted the weight and iron content of canola and maize shoots and roots. The present results demonstrated that delivering siderophores to the growing maize seedlings resulted in significant enhancement in biomass, relative to both full Hoagland solution and plants treated with Fe-free Hoagland solution conferring the stimulatory effect of *S. mundulus *siderophore. In this context, Kumari et al. [76] revealed that *Bacillus subtilis* DR2 siderophore acts as a biofertilizer, boosting *Coriandrum sativum* seed germination and vegetative growth. Similarly, Rangseekaew et al. [77] found that inoculation of tomato seedlings with *Dermacoccus barathri* MT2.1T and *D. profundi* MT2.2 Timproved plant growth by producing growth promoters such as siderophore synthesis, indole-3-acetic acid, and phosphate solubility.

Concerning Fe and K contents exhibited fast recovery of nutrients occurred in siderophore-supplemented maize seedlings. Breitkreuz et al. [14] found that during drought conditions, *Pseudomonas*-siderophores addition could improve soil nutrients via phosphate and potassium solubilization. Similarly, Vivas et al. [78] approved that under drought stress conditions the levels of potassium, nitrogen, and phosphorus rose following the inoculation of *Bacillus* sp. siderophore in lettuce.

## Conclusion

The outcome of this study highlights *Synechococcus mundulus* as a promising candidate for hydroxamate siderophore production. Optimization study including NaNO_**3**_ and iron concentrations in addition to pH level was successful for high siderophore production by *Synechococcus mundulus*. The significant enhancement in *Zea mays* seedlings’ growth performance in response to the inoculation with *Synechococcus mundulus* siderophore, highlighted its potential in managing maize growth under iron stressful condition. Findings of this study present novel visions of cyanobacteria producing siderophores as an ecofriendly alternative candidate to synthetic iron chelators and their role as cyanobacterial bio-amendments for ameliorating plant stress. Nevertheless, further research will be needed to investigate the effectiveness of cyanobacteria derived siderophores on other plant varieties at the field level to ensure long sustainability.

## Data Availability

No datasets were generated or analysed during the current study.
